# Recombinant production of bacterial toxins and their derivatives in the methylotrophic yeast *Pichia pastoris*

**DOI:** 10.1186/1475-2859-4-33

**Published:** 2005-12-07

**Authors:** Cemal Gurkan, David J Ellar

**Affiliations:** 1 Department of Biochemistry, University of Cambridge, 80 Tennis Court Road, Cambridge CB2 1GA, UK; 2 Department of Cell Biology, The Scripps Research Institute, 10550 North Torrey Pines Road, Mail Drop: MB6, La Jolla, CA 92037, USA

## Abstract

The methylotrophic yeast *Pichia pastoris *is a popular heterologous expression host for the recombinant production of a variety of prokaryotic and eukaryotic proteins. The rapid emergence of *P. pastoris *as a robust heterologous expression host was facilitated by the ease with which it can be manipulated and propagated, which is comparable to that of *Escherichia coli *and *Saccharomyces cerevisiae*. *P. pastoris *offers further advantages such as the tightly-regulated alcohol oxidase promoter that is particularly suitable for heterologous expression of foreign genes. While recombinant production of bacterial toxins and their derivatives is highly desirable, attempts at their heterologous expression using the traditional *E. coli *expression system can be problematic due to the formation of inclusion bodies that often severely limit the final yields of biologically active products. However, recent literature now suggests that *P. pastoris *may be an attractive alternative host for the heterologous production of bacterial toxins, such as those from the genera *Bacillus*, *Clostridium*, and *Corynebacterium*, as well as their more complex derivatives. Here, we review the recombinant production of bacterial toxins and their derivatives in *P. pastoris *with special emphasis on their potential clinical applications. Considering that *de novo *design and construction of synthetic toxin genes have often been necessary to achieve optimal heterologous expression in *P. pastoris*, we also present general guidelines to this end based on our experience with the *P. pastoris *expression of the *Bacillus thuringiensis *Cyt2Aa1 toxin.

## Review

With the advent of modern molecular biology, recombinant expression is now routinely used for the production of proteins of sufficient purity and quantity for their functional characterization and/or use in downstream applications. For example, heterologous expression systems have facilitated the development of recombinant vaccines against the bacterial toxins that are the causative agents of human diseases such as tetanus, botulism and cholera [1-4]. Concurrently, biosynthesis of novel proteins is feasible by engineering of recombinant DNA constructs that comprise of unrelated genes, which are also often from very diverse organisms. For instance, immunotoxins are therapeutic agents that are typically composed of DNA encoding a tumour-specific antibody fragment fused to a gene coding for a highly potent bacterial toxin or its subunits [5].

Despite their crucial roles in vaccine development, therapeutic applications, control of crop pests and disease vectors, as well as in basic research and functional characterization, heterologous expression of bacterial genes and their novel recombinant fusions may still pose unique challenges. For instance, bacterial toxins often have deleterious effects on the host cell physiology that may limit the final yields or may even exclude the use of certain recombinant expression systems altogether. Furthermore, bacterial genes may be unsuitable for heterologous expression in certain recombinant expression hosts due to the inherent features of the prokaryotic DNA sequences such as differences in codon usage and/or high A+T-content that may contain cryptic eukaryotic polyadenylation signals. Finally, if the bacterial toxins or their derivatives are destined for clinical use, more stringent recombinant production methods are necessary to ensure utmost purity, hence in some cases further limiting the choice of heterologous expression hosts. In this manuscript, we review the use of the *Pichia pastoris *(*P. pastoris*) expression system for the recombinant production of bacterial toxins and their derivatives, with special emphasis on their potential clinical applications.

### *P. pastoris *as a recombinant expression host

As a methylotrophic yeast, *P. pastoris *can use methanol as its sole carbon and energy source in the absence of a repressing carbon source [6,7]. The first step in the metabolism of methanol is its oxidation to formaldehyde by the enzyme alcohol oxidase (AOX) using molecular oxygen. In addition to formaldehyde, this reaction also generates hydrogen peroxide. To avoid hydrogen peroxide toxicity, methanol metabolism takes place within a specialised organelle called the peroxisome that sequesters the toxic by-products away from the rest of the cell. Since AOX has a poor affinity for oxygen, *P. pastoris *compensates by generating large amounts of the enzyme, which can accumulate to comprise up to 30% of total cell protein (tcp) during induction with methanol [8]. There are now a variety of vectors available that are mostly based on the powerful *AOX1 *promoter for the regulated overproduction of intracellular and secreted proteins in *P. pastoris *[9-11].

In contrast to the prokaryotic recombinant expression systems such as those based on *Escherichia coli *(*E. coli*), *P. pastoris *possesses eukaryotic features such as a secretory pathyway based on compartmentalized endomembranes, which is better equipped for post-translational modifications. Consequently, *P. pastoris *allows efficient secretory expression of complex recombinant proteins with correct intra- and inter-molecular disulphide bonds that do not require additional *in vitro *unfolding and refolding strategies. Furthermore, secreted expression in *P. pastoris *is a particularly attractive option because while it only secretes low-levels of endogenous proteins, it is capable of high-level secretion of the heterologously expressed proteins. *P. pastoris *can also be grown on simple, chemically-defined media, therefore secretion of the heterologous protein often becomes an effective purification step itself.

Other key features that contributed to the rapid emergence of *P. pastoris *as a robust recombinant expression host include: (1) the speed, ease and cost-effectiveness with which it can be manipulated and propagated compared to the other eukaryotic expression systems [12], (2) possession of tightly-regulated promoters, such as that of the *alcohol oxidase 1 *gene (*AOX1*), which is uniquely suited for the controlled expression of foreign genes [13,14], (3) synthesis of *N*-linked glycosylation moieties that resemble the mammalian high-mannose type [15], and (4) a strong preference for aerobic growth, a key physiological trait that greatly facilitates culturing at high cell densities relative to the fermentative yeast, *Saccharomyces cerevisiae *(*S. cerevisiae*). Indeed, *P. pastoris *can be grown up to 130 g·l^-1 ^dry cell weight on simple defined media [6]. Generally an immediate improvement in the percentage yield of heterologous protein expression is also observed on going from shake-flask cultures to bioreactor cultures [6].

### Heterologous expression of bacterial toxins and their derivatives in *P. pastoris*

As discussed in the previous section, *P. pastoris *is a popular recombinant expression host for a wide variety of prokaryotic and eukaryotic proteins [6,7]. Here we present a recent literature survey of the bacterial toxins and/or their derivatives that have been successfully produced in *P. pastoris *(Table 1).

**Table 1:** Bacterial toxins and their derivatives successfully expressed in *P. pastoris*. The bacterial toxin and the species it is originating from are given, along with brief notes on the specifics of the reported recombinant expression strategies.

**Bacterial toxin (*species*)**	**Remarks (expression culture type) [reference]**	**Final Yield^§^**
**TeNT(H_C_) **(*Clostridium tetani*)	intracellular expression^† ^of a synthetic^‡ ^gene encoding the tetanus toxin fragment C (B) [18]	12 g·l^-1 ^culture*
**BoNTA(H_C_) **(*Clostridium botulinum*)	intracellular expression^† ^of a synthetic gene^‡ ^encoding the heavy fragment C of the botulinum neurotoxin serotype A [BoNTA(H_C_)] (B) [19, 22-25]	770 mg·l^-1 ^culture
**BoNTB(H_C_) **(*Clostridium botulinum*)	intracellular expression† of a synthetic gene‡ encoding the heavy fragment C of the botulinum neurotoxin serotype B [BoNTB(H_C_)] (B) [1, 20, 24]	390 mg·kg^-1 ^cells
**BoNTC_1_(H_C_) **(*Clostridium botulinum*)	intracellular expression^† ^of a synthetic gene^‡ ^encoding the heavy fragment C of the botulinum neurotoxin serotype C_1 _[BoNTC_1_(H_C_)] (B) [25]	200–500 mg·kg^-1 ^cells
**BoNTE(H_C_) **(*Clostridium botulinum*)	intracellular expression of a synthetic gene^‡ ^encoding the heavy fragment C of the botulinum neurotoxin serotype E [BoNTE(H_C_)] (B) [25]	200–500 mg·kg^-1 ^cells
**BoNTF(H_C_) **(*Clostridium botulinum*)	intracellular expression of a synthetic gene^‡ ^encoding the heavy fragment C of the botulinum neurotoxin serotype F [BoNTF(H_C_)] (B) [21, 26]	240 mg·kg^-1 ^cells
**DT **(*Corynebacterium diphtheriae*)	secreted expression of a synthetic gene^‡ ^encoding the truncated diphtheria toxin (DT) fused to a bivalent antibody fragment (B) [30-33]	120 mg·l^-1 ^culture*
**BSP1 and BSP2 **(*Bacillus sphaericus*)	intracellular co-expression^† ^of synthetic genes^‡ ^encoding the mosquitocidal *B. sphaericus *polypeptides 1 and 2 (BSP1 and 2) (SF) [44]	<30% tcp*
**Cry2 **(*Bacillus thuringiensis*)	intracellular expression of Cry2 using the native bacterial DNA sequence (SF) [43]	N.D.
**Cyt2Aa1 **(*Bacillus thuringiensi*s)	intracellular expression of a synthetic gene^‡ ^encoding Cyt2Aa1 (SF) [34]	~1 mg·l^-1 ^culture*
**Cyt2Aa1 **(*Bacillus thuringiensis*)	synthetic gene^‡ ^encoding Cyt2Aa1 fused to a human scFv; secretory targeting resulted in ER-retention of the recombinant product (SF) [35]	10 mg·l^-1 ^culture
**Ace **(*Vibrio cholerae*)	secreted expression of the accessory cholera enterotoxin (Ace) using the native bacterial DNA sequence (SF) [28]	7 mg·l^-1 ^culture*
**Cef **(*Vibrio cholerae*)	secreted expression of Chinese hamster ovary (CHO) cell-elongating factor (Cef) using the native bacterial DNA sequence (SF) [29]	N.D.
**CTB **(*Vibrio cholerae*)	secreted co-expression of the cholera toxin subunit B (CTB) and CTB-viral antigen fusion protein using the native bacterial DNA sequence (SF) [4]	N.D.
**LTB **(*Escherichia coli*)	secreted expression of the heat-labile enterotoxin subunit B (LTB) using the native bacterial DNA sequence (SF) [27]	8 mg·l^-1 ^culture
**LTB **(*Escherichia coli*)	intracellular expression of a LTB and a viral antigen fusion protein using the native bacterial DNA sequence (SF) [27]	N.D.

^†^Using *P. pastoris *transformants that are selected for the presence of multiple copies of the chromosomally-integrated heterologous expression cassettes; ^‡^synthetic gene with optimal *P. pastoris *codon usage and reduced A+T-content; ^§^only the highest final yields are reported in this table; *estimated total expression; (SF): shake-flask culture, (B): bioreactor culture; N.D.: no data available; ER: the endoplasmic reticulum.

Experience with the recombinant production of the *Clostridium *neurotoxin fragments in *P. pastoris *provides good examples for the typical problems encountered with the heterologous expression of bacterial toxins in this yeast and the subsequent high yields attainable once these problems are properly addressed. *Clostridium botulinum *is the causative agent of botulism, which is a severe neuroparalytic disease brought about by one of the seven antigenically distinct neurotoxin (BoNT) variants (A, B, C_1_, D, E, F and G) produced by this bacterium [1-3]. Similarly, *Clostridium tetani *produces tetanospasmin or the tetanus neurotoxin (TeNT) that causes the spastic paralysis condition associated with the tetanus disease. Both TeNT and the BoNT variants are potent exotoxins that are initially synthesized as a single polypeptide chain that typically undergoes subsequent proteolytic processing into a heterodimer of heavy and light chains bound together by a disulphide bond. In both TeNT and the BoNT variants, the carboxyl-terminal domain of the heavy chain (H_C_) is non-toxic and associated with binding to specific receptors present on the target nerve cells, and since it is antigenic, it has been exclusively used for vaccine development [1,2]. Currently, a pentavalent botulinum toxoid from natural sources composed of variants A through E and a toxoid of variant F are used to immunize at-risk individuals, such as scientists and health care workers that handle BoNT or armed forces personnel that may be subject to weaponized forms of the bacterial toxin [2,3]. However, this strategy has many shortcomings because: (1) *C. botulinum *produces only low-levels of the most toxin variants, (2) large-scale production is very costly and dangerous, requiring dedicated facilities in accordance with the current Good Manufacturing Practices, (3) the final products are whole toxins that are only partially homogenous, which may in turn influence immunogenicity or reactivity of the vaccine, and (4) the toxoiding process involves the use of chemicals such as formaldehyde and thimerosal that are still present in the final product formulation, hence rendering it reactogenic [2,3]. Consequently, there is a great demand for the development of a new generation of recombinant vaccines that would alleviate many of the problems associated with the current toxoid formulations.

Recombinant tetanus neurotoxin fragment C [TeNT(H_C_)] was the first bacterial toxin that was successfully expressed in *P. pastoris *[16]. Earlier attempts at heterologous TeNT(H_C_) expression in *E. coli *and *S. cerevisiae *necessitated the use of synthetic genes due to the unfavorable codon bias of the A+T-rich *C. tetani *DNA sequence and the presence of cryptic polyadenylation signals that led to premature mRNA transcript termination in yeast [17]. Following a similar approach, Clare *et al. *used a synthetic gene with altered codon usage that had a substantially reduced A+T-content to achieve recombinant production of TeNT(H_C_) in *P. pastoris *with final yields as high as 12 g per liter of bioreactor culture [18]. Recombinant production in *P. pastoris *of BoNT variants was also very successful when using synthetic genes that were optimized for heterologous expression in this yeast [1,2,19-26]. As in the case of TeNT(H_C_) [18], heterologous expression of BoNTA(H_C_), B(H_C_), and E(H_C_) in *P. pastoris *was also attempted by secretory targeting of the recombinant products [1,2]. However, in both cases the recombinant proteins secreted into the culture medium were glycosylated due to the presence of fortuitous *N*-linked glycosylation sites in the prokaryotic primary amino acid sequences. This glycosylation rendered them immunologically inactive, hence unfit for vaccine development unless a costly *in vitro *deglycosylation step was carried out [1,2,18]. Accordingly, both TeNT(H_C_) and the BoNT(H_C_) variants are now exclusively produced by intracellular heterologous expression in *P. pastoris *(Table 1). For vaccine development, production in *P. pastoris *offers additional advantages over *E. coli *in avoiding the formation of inclusion bodies during heterologous expression and eliminating the potential presence of bacterial endotoxins requisite to achieve Food and Drug Administration licensure [2,3].

*P. pastoris *also proved very useful in the development of vaccines for the heat-labile enterotoxin (LT) of *E. coli *and the cholera toxin (CT) of *Vibrio cholerae*, which both cause diarrhea in humans [4,27]. Both LT and CT have a hetero-hexameric structure consisting of a toxic A subunit and five non-toxic B subunits that function in binding to the target cells. The LT subunit B (LTB) was successfully expressed in *P. pastoris *using the bacterial gene and efficiently secreted into culture medium in a native-like pentameric form that was biologically active and immunogenic [27]. Fingerut *et al. *also reported intracellular expression in *P. pastoris *of a genetic fusion of LTB with a viral antigen to demonstrate the adjuvant activity of recombinant LTB produced in the methylotrophic yeast [27]. Similarly, CT subunit B (CTB) and a genetic fusion of CBT with a viral vaccine antigen were successfully co-expressed in *P. pastoris *using the native bacterial CBT gene [4]. This allowed efficient co-secretion of the recombinant CBT and CBT fusion proteins into the culture medium in a biologically active hetero-pentameric form, which could then be purified by a single-step affinity-tag based chromatography strategy. Other *V. cholerae *toxins also successfully expressed in *P. pastoris *are the accessory cholera toxin (Ace) and the Chinese hamster ovary (CHO) cell-elongating factor (Cef) [28,29]. Despite having a key role in *V. cholerae *pathogenesis, the accessory cholera toxin (Ace) is produced only at low levels by it natural host, which initially hampered its further characterization [28]. While recombinant production of Ace in *E. coli *was not feasible due to inherent toxicity effects to the host cells, Trucksis *et al. *reported subsequent success using the *P. pastoris *expression system, where secreted enterotoxin could be purified to homogeneity in a biological active form and at levels as high as 7 mg·l^-1 ^culture [28].

Bacterial toxins have further clinical applications, such as in the development of novel therapeutic agents. These include immunotoxins (ITs) comprised of a potent bacterial toxin that is recombinantly fused to a cell-binding ligand such as an antibody fragment specific for tumor cells [5]. Recombinant expression of ITs can be particularly challenging due to the deleterious effects of the toxin moiety on the host cell physiology and/or the presence of multiple disulphide bonds in the antibody fragment moiety that are requisite for its function. However, recent literature suggests that the *P. pastoris *expression system might be an attractive alternative for recombinant IT production. For example, Woo *et al*. reported successful fine-tuning of the *P. pastoris *expression system for the production of a recombinant IT based on a truncated version of the diphtheria toxin (DT) [30-32]. This strategy necessitated the construction of a synthetic gene optimized for *P. pastoris *expression that encoded the first 390 amino acids of the DT toxin (DT390) previously shown to be the minimum DT truncate suitable for IT production [30]. The multi-domain DT390-based IT could be efficiently secreted by *P. pastoris *in a biologically active form and at yields as high as 10 mg·l^-1 ^of shake-flask culture [30]. Notably, *P. pastoris *proved to be a particularly suitable recombinant expression host in this case as it has a higher tolerance for DT toxicity compared to *S. cerevisiae *and other eukaryotes. While the introduction of a DT resistant mutation into the chromosomal EF-2 locus of *P. pastoris *did not help to further increase the final yields of biologically active IT, secreted expression levels as high as 120 mg·l^-1 ^culture were eventually achieved using a bioreactor and empirically optimized methanol induction conditions [31-33].

We have recently reported the successful recombinant production in *P. pastoris *of the Cyt2Aa1 δ-endotoxin from the *Bacillus thuringiensis *(*B. thuringiensis*) subspecies *kyushuensis*, as well as that of a membrane-acting Cyt2Aa1-based IT [34,35]. *B. thuringiensis *is a ubiquitous aerobic, gram-positive bacterium that is best known for its crystalline δ-endotoxin inclusions produced during sporulation [36]. These δ-endotoxins are pore-forming proteins with very specific larvicidal activities for insects in the order of Lepidoptera, Coleoptera and Diptera. All active δ-endotoxins belong to either the Cry or Cyt family of toxins that share very little amino acid sequence identity but are both initially produced as protoxins that need to be solubilized at the appropriate pH prior to activation by proteolytic processing. Cyt toxins are smaller than the Cry toxins and are further distinguished from the latter by: (1) their highly specific mosquitocidal activity *in vivo*, (2) their broad cytolytic activity to a variety of invertebrate and vertebrate cells *in vitro *after solubilisation and activation by proteolytic processing, and (3) their ability to spontaneously insert into membranes containing zwitterionic phospholipids with unsaturated acyl chains [37-39]. This unique combination of features makes Cyt toxins highly suitable for the development of membrane-acting ITs, an alternative idea in the field that was initially explored in our laboratory using chemical conjugation strategies [40,41].

Considering that recombinant production methods would provide more homogenous Cyt-based ITs compared to the chemical conjugation strategies, subsequent attempts in our laboratory were based on the use of the *E. coli *expression system. However, this strategy led to only limited success due to the invariable formation of inclusion bodies in this prokaryotic expression host, which in turn limited the final yields of biologically active Cyt-based ITs. Consequently, we next attempted the recombinant production of Cyt2Aa1-based ITs in *P. pastoris *using the native bacterial gene. However, as it has been the case for the majority of other bacterial toxins that are also encoded by A+T-rich genes (Table 1), recombinant production of Cyt2Aa1 and Cyt2Aa1-based ITs in *P. pastoris *necessitated *de novo *design and construction of a synthetic toxin gene that was optimized for heterologous expression in this yeast [34,35]. Since *de novo *design and construction of synthetic genes is often a prerequisite for achieving heterologous expression of bacterial toxins in *P. pastoris *(Table 1), we present general guidelines to this end in the next section based on our experience with the heterologous Cyt2Aa1 expression in this yeast.

In contrast to the intracellular expression of the native bacterial gene in *P. pastoris*, that of the synthetic gene led to the recombinant production of the Cyt2Aa toxin, albeit severe product toxicity effects were observed [34]. Similar toxicity effects were also observed with the intracellular expression of the Cyt2Aa1-based IT in the same heterologous expression host, which could be largely alleviated by the secretory targeting of the recombinant product. While the Cyt2Aa1-based IT failed to be secreted from the *P. pastoris *cells, secretory targeting proved beneficial in this case since it sequestered the deleterious recombinant product from the yeast cytosol, where a wide range of organelles would otherwise be prone to Cyt2Aa1-based membrane damage [35]. Instead, the recombinant Cyt2Aa1-based IT accumulated to high-levels in the yeast endoplasmic reticulum, where the high local Ca^2+ ^concentration in this organelle is expected to be inhibitory to the basic Cyt2Aa1 toxin activity [35,42]. Furthermore, secretory targeting allowed proper formation of the disulphide bonds requisite for the function of the cell-binding domain of the recombinant Cyt2Aa1-based IT, which could then be recovered in a biologically active form at 10 mg·l^-1 ^culture by a chaotropic denaturation step that was followed with an on-the-column refolding strategy [35]. While the final yield of biologically active Cyt2Aa1-based IT could be potentially increased through the selection of multi-copy integrants of the recombinant expression cassette and/or large-scale bioreactor cultures (Table 1), we did not find this to be necessary for the purposes of our project, which was the development of an *in vitro *model system to test the potency of Cyt2Aa1-based ITs. However, it has also not escaped our attention that Cyt2Aa1-expressing *P. pastoris *cells can have further potential use in the control of disease vectors, as has proved to be the case for the last two examples of *P. pastoris *heterologous expression that we present below.

Recombinant expression in *P. pastoris *of the *B. thuringiensis *insecticidal Cry2 toxin has also been described using the native bacterial gene [43]. In addition, high-level (up to 30% tcp) *P. pastoris *co-expression of two biologically active *B. sphaericus *mosquitocidal proteins BSP1 and BSP2 was reported using synthetic genes that were optimized for heterologous expression in this yeast [44]. Here, *P. pastoris *cells expressing the *B. sphaericus *insecticidal proteins were heat-killed without a significant reduction in the biological activity of the recombinant toxins and then fed to Dipteran larvae, which are filter-feeders that usually find yeast cells palatable [44]. This strategy has a minimal risk of releasing the heterologous toxin gene into environment since it would be integrated into the yeast genome unlike the autonomous plasmids used for heterologous expression in *E. coli*.

### Design and de novo synthesis of bacterial genes for optimal expression in *P. pastoris*

There are now various commercial services available that offer total gene synthesis at competitive prices. However, it is also possible to design and construct any given DNA sequence using well established protocols [30,34,44-46]. Here we present as an example, the strategy that we have successfully used for the design and *de novo *construction of a synthetic gene coding for the *B. thuringiensis *Cyt2Aa1 toxin that was optimized for expression in *P. pastoris *[34,35].

As discussed previously, our initial attempts at heterologous Cyt2Aa1 expression in *P. pastoris *were unsuccessful due to inherent problems with the eukaryotic expression of the bacterial gene. This was attributed to the high A+T-content of the native Cyt2Aa1 gene containing cryptic polyadenylation sites that resulted in premature transcription termination in yeast [17,18]. To achieve optimal heterologous expression in *P. pastoris*, we designed a synthetic gene based on the primary amino acid sequence of the proteinase K-activated form of the Cyt2Aa1 toxin [34]. To this end, the overall A+T-content of the bacterial gene was systematically reduced by changing its codon usage to that preferred by *P. pastoris *(Table 2). Our manual selection largely favoured the most-preferred *P. pastoris *codons, but in certain instances the second-most preferred codons were selected instead to ensure an overall reduction in the A+T-content of the resulting DNA sequence. This strategy resulted in the reduction of the A+T-content from ~70% to 50%, while retaining only 18.5% of the original codon usage. Furthermore, our synthetic gene design also ensured that the initial 50–75 nucleotides of the corresponding mRNA would be free of stable secondary structures, especially in the vicinity of the translation initiation codon [47], and the overall DNA sequence would not contain the restriction enzyme sites that would be used during the subsequent cloning strategies, *etc*. Rational design of the synthetic gene was facilitated by the use of the Genetics Computer Group (GCG) software package (Wisconsin Package version 10.2-UNIX, Madison, WI) [48], especially the programs MFold, PlotFold and Map. A Kozak consensus translation initiation sequence for yeast was also introduced into synthetic gene to ensure its efficient heterologous expression in *P. pastoris *[49]. Finally, *de novo *synthesis of the synthetic Cyt2Aa1 gene was readily achieved by a recursive PCR strategy that used overlapping oligonucleotides representing the partial sequence of the sense and anti-sense strands of the proposed DNA sequence [34,35,45]. Briefly, all oligonucleotides were designed to be between 57–71 nucleotides and to have a similar theoretical melting temperature (52–56°C), as well as a 19–23 bp overlap at their 3'-end. To ensure the specificity of each pairing and the absence of any undesirable secondary structures, all oligonucleotide selections were extensively analysed by GCG FastA and Stemloop programs [48,50]. The mutual extension of the overlapping oligonucleotides produces longer double-stranded products, and ultimately the full-length synthetic gene construct, which is then amplified by the 5'-outermost flanking primers [34].

**Table 2:** *P. pastoris *codon preference. This codon preference table was compiled from literature and is based on highly expressed genes in *P. pastoris*, as well as those in other yeast species such as *S. cerevisiae *[44, 51-53].

**Amino acid**	**1^st ^preference**	**2^nd ^preference**	**Amino acid**	**1^st ^preference**	**2^nd ^preference**
Ala (A)	GCT	GCC	Leu (L)	TTG	CTT/CTG
Arg (R)	AGA	CGT	Lys (K)	AAG	AAA^§^
Asn (N)*	AAC	AAT	Met (M)	ATG	-
Asp (D)	GAC	GAT	Phe (F)	TTC	TTT^§^
Cys (C)*	TGT	TGC	Pro (P)	CCA	CCT
Gln (Q)^†^	CAA	CAG	Ser (S)^†^	TCT	TCC
Glu (E)^‡^	GAG	GAA	Thr (T)^†^	ACT	ACC
Gly (G)	GGT	GGA	Trp (W)	TGG	-
His (H)*	CAC	CAT	Tyr (Y)*	TAC	TAT
Ile (I)^†^	ATT	ATC	Val (V)	GTT	GTC

^†^Amino acids for which there is a minimal bias between the first and second-most preferred codons; ^‡^rare amino acids constituting a major discrepancy between the *P. pastoris *and *S. cerevisiae *codon preferences; *amino acids with a very high bias for the first preference codon. Other general trends observed with yeast codon preferences are as follows: (1) ^§^codons that contain 100% G, C, A or T are best avoided, (2) there is a strong avoidance of side-by-side GC base pairs in codon-anticodon interactions, (3) there are three codons used for translational termination, which are used with the frequency TAA > TAG > TGA, and (4) the *S. cerevisiae *consensus sequence for translation initiation context is **A/Y A A/U A AUG UCU **(where Y is a pyrimidine base, C or T), however it has been shown to have only a moderate effect on translation [51, 53, 54].

## Conclusion

*P. pastoris *is a robust recombinant expression host that has also seemingly emerged as an alternative heterologous expression host for a variety of bacterial toxins and their derivatives. In particular, secretory targeting is an advantageous strategy for the recombinant production of toxins and/or their derivatives that require proteolytic processing and/or proper disulphide bond-formation for their activity. In this respect, *P. pastoris *may be better suited than the *E. coli*- and *S. cerevisiae*-based expression systems and it may also allow higher yields of biologically active recombinant protein as it can be grown to high cell densities under aerobic conditions. As in the case of *B. thuringiensis *Cyt2Aa1 toxin that is not secreted by the native host, secretory targeting of the fusion proteins may also help alleviate product toxicity effects on the *P. pastoris *cells. However, undesirable glycosylation of the secreted bacterial toxins may need to be addressed when using this strategy, such as by: (1) introducing silent mutations to remove cryptic glycosylation sites present in the prokaryotic primary amino acid sequence, (2) although it may be cost-prohibitive for large-scale applications, *in vitro *enzymatic deglycosylation can be carried out, or alternatively, (3) intracellular expression of the toxin can be attempted. A further potential problem that is often encountered during heterologous expression of the bacterial toxins in *P. pastoris *centers on differences in the codon bias of the A+T-rich prokaryotic toxin genes that can minimize or even preclude the recombinant production of the full-length proteins. However, there are now many examples in the literature on the successful use of *de novo *synthesized bacterial genes that are optimized for heterologous expression in this yeast.

## List of abbreviations used

AOX: alcohol oxidase; *AOX1*: *P. pastoris *major alcohol oxidase gene; tcp: total cell protein; BoNT and TeNT, botulinum and tetanus neurotoxins, respectively; BoNT(H_C_) and TeNT(H_C_), the carboxyl-terminal domain of the heavy chain fragment of the botulinum and tetanus neurotoxins, respectively; LT and CT: the heat-labile *E. coli *enterotoxin and the *V. cholerae *toxin, respectively; LTB and CTB: B subunit of the heat-labile *E. coli *enterotoxin and the *V. cholerae *toxin, respectively; CHO: Chinese hamster ovary; Cef: cell-elongating factor; IT: immunotoxin; DT: diphtheria toxin; DT390: truncated version of DT corresponding to the first 390 amino acid residues.
